# Revisited role of the placenta in bile acid homeostasis

**DOI:** 10.3389/fphys.2023.1213757

**Published:** 2023-07-21

**Authors:** Edgar Ontsouka, Mariana Schroeder, Christiane Albrecht

**Affiliations:** Institute of Biochemistry and Molecular Medicine, University of Bern, Bern, Switzerland

**Keywords:** bile acid signaling, bile acid synthesis, fetal development, placenta, pregnancy

## Abstract

To date, the discussion concerning bile acids (BAs) during gestation is almost exclusively linked to pregnancy complications such as intrahepatic cholestasis of pregnancy (ICP) when maternal serum BA levels reach very high concentrations (>100 μM). Generally, the placenta is believed to serve as a protective barrier avoiding exposure of the growing fetus to excessive amounts of maternal BAs that might cause detrimental effects (e.g., intrauterine growth restriction and/or increased vulnerability to metabolic diseases). However, little is known about the precise role of the placenta in BA biosynthesis, transport, and metabolism in healthy pregnancies when serum BAs are at physiological levels (i.e., low maternal and high fetal BA concentrations). It is well known that primary BAs are synthesized from cholesterol in the liver and are later modified to secondary BA species by colonic bacteria. Besides the liver, BA synthesis in extrahepatic sites such as the brain elicits neuroprotective actions through inhibition of apoptosis as well as oxidative and endoplasmic reticulum stress. Even though historically BAs were thought to be only “detergent molecules” required for intestinal absorption of dietary fats, they are nowadays acknowledged as full signaling molecules. They modulate a myriad of signaling pathways with functional consequences on essential processes such as gluconeogenesis -one of the principal energy sources of the fetus- and cellular proliferation. The current manuscript discusses the potential multipotent roles of physiologically circulating BAs on developmental processes during gestation and provides a novel perspective in terms of the importance of the placenta as a previously unknown source of BAs. Since the principle “not too much, not too little” applicable to other signaling molecules may be also true for BAs, the risks associated with fetal exposure to excessive levels of BAs are discussed.

## 1 General aspects of bile acid synthesis and regulation

**FIGURE 1:**
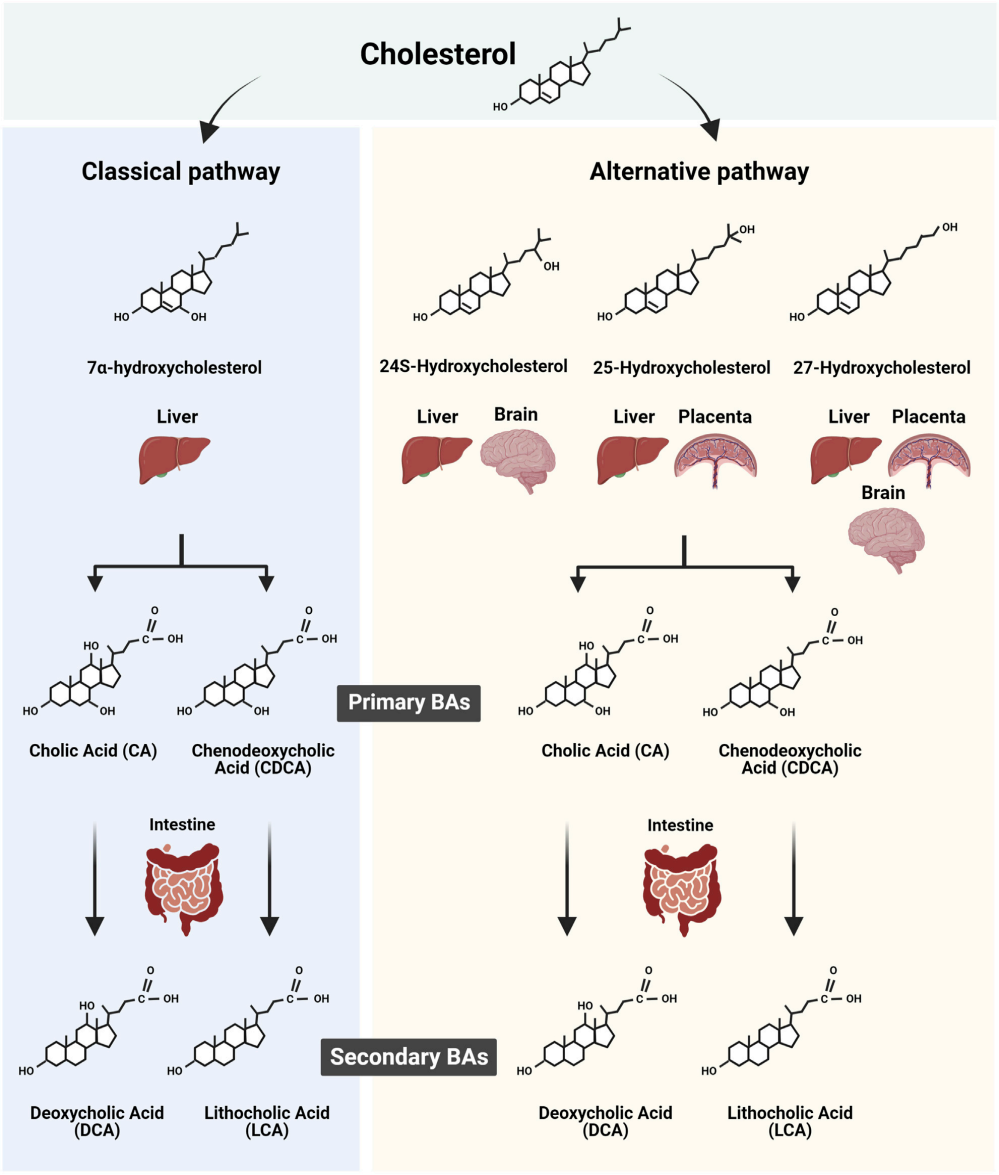
Scheme summarizing the biosynthesis of primary and secondary bile acids (BAs) in human tissues. The two major routes of BA synthesis, namely, the classic (or neutral) and the alternative (or acidic) pathways, are illustrated. The cholesterol derivates implicated in the classic and alternative BA synthesis pathways in the liver, brain, and potentially placenta are shown in conjunction with the resulting primary BAs synthesized. In addition, the corresponding secondary BAs resulting from the intestinal modification of primary BA are depicted. Figure created with BioRender.com.

The primary bile acids (BAs) in humans, cholic acid (CA) and chenodeoxycholic acid (CDCA) are cholesterol derivatives produced in the liver through multistep synthetic processes involving several specific enzymes. These enzymes are hosted in subcellular compartments including peroxisomes. The BAs, bearing a pentanoic acid side chain and one to three hydroxyl groups are positioned at α3, α7, and α12 of the cholane, and are synthesized through two major routes, the classic (or neutral) pathway and the alternative (or acidic) pathway (Russell and Setchell, 1992; Russell, 2003; Chiang, 2009) (Figure 1). The classical pathway is initiated by the rate-limiting enzyme cytochrome P450 (CYP)7A1 and ends with the synthesis of CA. The alternative pathway, controlled among others by CYP27A1, produces CDCA. The alternative BA pathway begins with hydroxylation of the side chain of cholesterol, producing an oxysterol. The latter is then hydroxylated at the 7α-position by an oxysterol 7α-hydroxylase. Of note, depending on the organ, different pathways such as sterol 27-hydroxylase (CYP27A1, in the liver) and/or cholesterol 25-hydroxylase (CH25H, also in the liver) and/or cholesterol 24-hydroxylase (CYP46A1 and CH24H in liver and brain) initiate BA biogenesis (Figure 1). The resulting intermediate molecules, like 3β-hydroxy-5-cholenoic acid, can undergo 7α-hydroxylation and multistep conversions that end with the production of CDCA. The BA synthetic pathways are also present in other highly metabolic organs such as the brain (Parker et al., 2020; Monteiro-Cardoso et al., 2021) and the murine and human placenta (Russell, 2003; Ontsouka et al., 2023). Regardless of the synthetic pathway implicated, the final steps in BA biogenesis involve the enzymatic actions of BA-CoA synthase and BA CoA: amino acid *N*-acyltransferase (BAAT). These enzymes promote the conjugation (or amidation) of CA and CDCA with taurine to form tauro-CA (TCA) and tauro-CDCA (TCDCA), respectively. In addition, some primary BAs are conjugated with glycine to form glyco-CA (GCA) and glyco-CDCA (GCDCA), respectively (Russell, 2003; Chiang and Ferrell, 2019).

The amino acid-conjugated BAs exhibit increased hydrophilicity and enhanced transmembrane transport, resulting in reduced cellular cytotoxicity. Within the intestinal lumen, primary BAs are deconjugated and dehydroxylated at the 7α-position under the enzymatic action of intestinal bacterial microflora (Niefan et al., 2015), giving rise to secondary BAs species (Begley et al., 2005; Ridlon et al., 2016; Sender et al., 2016). Specifically, internal modifications of CA and CDCA convert them into deoxycholic acid (DCA) and lithocholic acid (3α-hydroxy-5β-cholanoic acid; LCA), respectively. It is well documented that both primary and secondary BA species present in the intestine, are efficiently absorbed by the intestinal lumen, recycled back to the liver via the portal vein, and re-secreted into bile, a process known as enterohepatic circulation. This closed circuit allows only a small portion of BAs to bypass the enterohepatic circulation to enter the blood circulation, keeping the BA blood content relatively low (Hofmann, 2009).

The regulation of BA biogenesis is, among other factors, subject to diurnal variations. In rats and mice, BA synthesis peaks during the dark phase when they feed the most (Sundseth and Waxman, 1990), while in humans it peaks during daytime (Poole and Duane, 1988; Gälman et al., 2005). The rhythmicity of the hepatic BA synthesis is transcriptionally regulated (Sundseth and Waxman, 1990). Moreover, BA metabolism might be affected by autocrine, paracrine, and/or endocrine actions of other important molecules, such as estrogen, progesterone and their metabolites, and melatonin, all these being intrinsically produced by the placenta (Iwasaki et al., 2005; Lanoix et al., 2012; Karahoda et al., 2021). Estrogen, progesterone, and their metabolites increase hepatic BA production and reduce bile canaliculi release during pregnancy, leading to elevated total BA concentrations in the peripheral blood of pregnant women. Maternal melatonin blood levels gradually increase during pregnancy (Iwasaki et al., 2005; Lanoix et al., 2012), and melatonin seems to act as a protective molecule that balances oxidative effects and apoptosis caused by diverse effectors, including high circulating BAs (Zagrean et al., 2019; Chuffa et al., 2020; Gomes et al., 2021; Zhu et al., 2021). Melatonin freely crosses the biological membranes, acting intracellularly by modulating various signaling pathways (Chuffa et al., 2019; Zagrean et al., 2019). Unlike hepatic BA production in humans, melatonin rhythmically released by the pineal gland shows its highest level during the night. From a physiological point of view, it would be interesting to evaluate in future studies if BA biosynthesis in the liver and the placenta is rhythmically synchronized. Interestingly, one study in rats described concomitant increases of the BA contents in the maternal and fetal sera, in the liver, and in the placenta (Herraez et al., 2014), but the source of these BA was not investigated.

## 2 Placenta and bile acids

BAs exhibit marked effects on placental functions (Sepúlveda et al., 1991; Egan et al., 2012; Lofthouse et al., 2019) as well as on fetal development (De Aguiar Vallim et al., 2013; Li et al., 2020). Thus, for instance, exposure to high concentrations of BAs, particularly to LCA, exerted a constrictive effect on chorionic veins in humans (Sepúlveda et al., 1991; Lofthouse et al., 2019). Moreover, in the perfused cotyledon an increase in pressure and the constriction of chorionic arterial vessels were observed in the presence of taurine-conjugated CA (Lofthouse et al., 2019). However, the precise source of BAs acting on the placenta is not clearly described. The clarification of this important aspect implies the need to examine the probability and relevance of locally synthesized BAs, which could not only act on the placenta and surrounding tissues but may also impact fetal development and pregnancy outcomes.

### 2.1 Physicochemical effects of bile acids in the placenta

From the original discovery in 1848 by Heinrich Otto Wieland until their identification as hormone-like molecules (Maruyama et al., 2002; Kawamata et al., 2003), BAs have been historically known as “detergent molecules” thanks to their physicochemical properties. The physicochemical effects of BAs are largely dependent on their hydrophobicity, which is also an important determinant of their toxicity. It is known that hydrophobic BAs can damage cell membranes and promote oxidative stress, necrosis and apoptosis, while hydrophilic BA species protect against oxidative stress and inhibit apoptosis (Perez and Britz, 2009). This feature is determined by the number, position, and orientation of the hydroxyl groups, and the amidation (i.e., conjugation) at position C-24. Thus, the magnitude of their hydrophobicity is UDCA (ursodeoxycholic acid) < CA < CDCA < DCA < LCA (Thomas et al., 2008). The sulfation of BAs leads to the production of water-soluble BA species (Alnouti, 2009). Placental sulfatase as well as sulfotransferase activities have been reported, allowing the placenta to convert unconjugated steroids (e.g., BAs and estrogens), into their sulfated forms and *vice versa* (Stanley et al., 2001; Miki et al., 2002; Dongning et al., 2004). These sulfatase and sulfotransferase activities of the placenta probably constitute a metabolic barrier to the transfer of potentially toxic hydrophobic BAs from the maternal to the fetal circulation.

### 2.2 Receptor-mediated bile acid effects in the placenta

**FIGURE 2:**
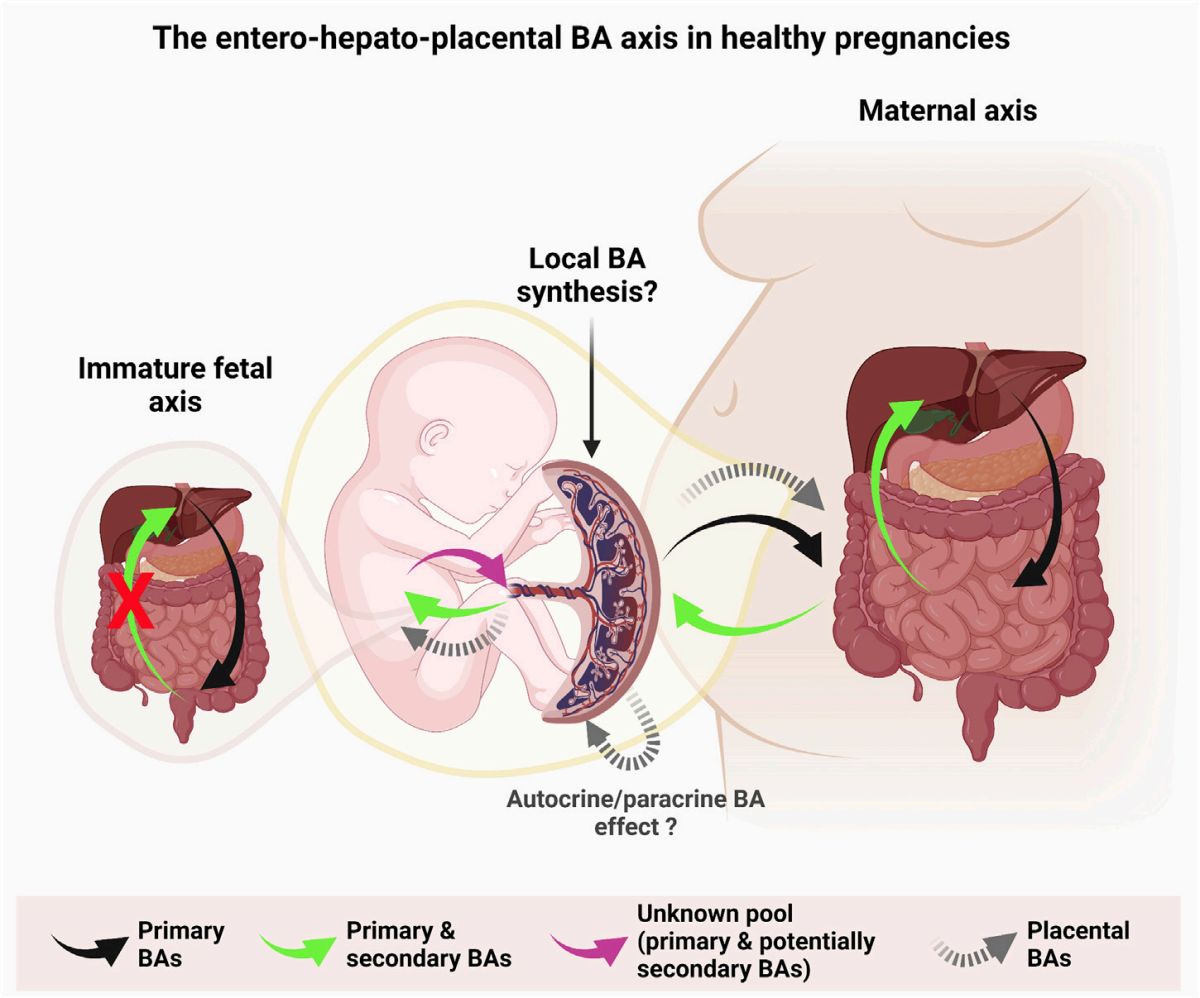
Model of the proposed entero-hepato-placental bile acid (BA) axis during a healthy pregnancy. The proposed action of this axis relies on the role of BAs as signaling molecules. Primary BAs are synthesized by the maternal liver, conjugated and unconjugated to an amino acid residue (in humans, glycine, or taurine) and are released into the maternal intestine where they can be modified to secondary BA by enzymatic activities of the maternal intestinal microbiota (right, black arrow). Circulating maternal primary and secondary BAs are efficiently transported across the intestine and recycled back to the liver (right, green arrow). A small proportion (5–10%) of the recycled intestinal BAs is directed towards the periphery, thereby reaching the placenta (right, green arrow), which they cross to enter the fetal circulation (center, green arrow). In turn, primary BAs synthesized by the mature fetal liver, conjugated and unconjugated to an amino acid residue and released into the fetal intestine (left, black arrow), do not undergo modifications in the fetal intestine since the latter is not yet colonized by specific intestinal microbiota (left, green arrow with symbol X) and the enterohepatic circulation is not yet functional. Therefore, the fetus-borne primary BAs and the potentially recycled maternally originating BAs (center, pink arrow) are directed towards the placenta where they are expelled into the maternal circulation. In addition, we propose that the placenta may locally synthesize primary BAs which are added to the overall BA pool and act on the placental, maternal, and fetal compartments (center, dashed arrows). The placenta allows the transport and action of the BA species in both directions, potentially including those that are synthesized *in situ*. Figure created with BioRender.com.

BAs are nowadays fully acknowledged as signaling molecules that elicit functional effects by interacting with specific cell surface G-protein coupled receptors (GPCRs) expressed on the apical and basal sides of the syncytiotrophoblast (Figure 2). BAs are not only natural ligands for cell surface GPCRs, but also interact with nuclear receptors, such as farnesoid X receptors (FXRs), pregnane X receptor (PXR), vitamin D receptor (VDR), retinoid X receptors (RXR), and the constitutive androstane receptor (CAR). Receptor-mediated effects of BAs play key roles in the regulation of lipid, glucose, and energy metabolism (De Aguiar Vallim et al., 2013; Li et al., 2020), processes that are crucial for adequate placental function.

The G protein-coupled bile acid receptor 1 (GPBAR1), also known as Takeda G protein-coupled receptor 5 (TGR5), is activated by both primary and secondary BAs. It is mainly responsive to LCA, with the following affinity rank: LCA > DCA > CDCA > CA > UDCA (Maruyama et al., 2002; Kawamata et al., 2003; Schaap et al., 2014). In the human placenta, GPBAR1/TGR5 protein was identified in some regions of the trophoblasts at term, as well as in fetal macrophages. Upon BA-induced activation, GPBAR1/TGR5 mediates important functions, including smooth muscle contractility, inflammatory responses, regulation of mucosal permeability, and alteration of insulin release (González-Mariscal et al., 2008; Rajagopal et al., 2013; Guo et al., 2016; Pathak et al., 2018; Zhan et al., 2020). At the mRNA level, *GPBAR1/TGR5* was detected in several tissues including the placenta and brain (Kawamata et al., 2003; Keitel et al., 2015; Guo et al., 2016; Klindt et al., 2019).

Sphingosine-1-phosphate (S1P), a natural ligand of the sphingosine-1-phosphate receptors (SP1R), is abundantly present in red blood cells, vascular endothelial cells, and activated platelets (Yatomi et al., 2001; Ryu et al., 2002). Expression of S1PR subtypes 1, 3, and 5 at the mRNA level, of S1PR_1_ at the protein level (Dobierzewska et al., 2016), and the presence of S1P kinase have been reported in the human placenta (Johnstone et al., 2005). The activation of S1PR_1-5_ is associated with the modulation of important physiological processes, such as vascular development during embryogenesis, wound repair, and cancer metastasis (Bartke and Hannun, 2009). S1PR subtypes 1 and 3 also appear to be reduced in preeclampsia (Dobierzewska et al., 2016). S1P and its synthesizing enzyme sphingosine kinase (SPHK) seem to have an angiogenic bioactive role in the process of trophoblast differentiation and invasion (Johnstone et al., 2005; Singh et al., 2012; Zhang et al., 2013) and placental angiogenesis (Mizugishi et al., 2007). Thus, the functional effects of BA might involve the activation of the S1PRs and influence placental and fetal development by improving nutrient delivery to the fetus.

Previous data further indicate that muscarinic acetylcholine receptors (M), a subfamily of GPCRs that regulate numerous fundamental functions of the central and peripheral nervous system, exhibit a high binding affinity for BAs (Raufman et al., 2002; Ibrahim et al., 2018; Ticho et al., 2019). The M subtype 2 is the predominant form found in human term placenta (Colmenero Salas et al., 2009), but M_1_-M_4_ were also detected (Tayebati et al., 1997). The functional consequences of the BA-induced activation of the Ms include the regulation of blood flow and fluid volume in placental vessels, the opening and closing of trophoblastic channels, the induction of the contractile properties of myofibroblasts, the facilitation of amino acid transport across the placenta, the release of placental hormones, and modulation of the formation of myometrial and placental prostaglandins in human parturition (Sastry, 1997).

### 2.3 Transplacental transport of bile acids to the sites of action

BA species are transported to their sites of action by membrane proteins of the ATP-binding cassette (ABC) transporter and solute carrier (SLC) families. Some of these membrane transporters, such as the organic anion transporting polypeptide (OATP)1B1/SLCO1B1, OATP1B3/SLCO1B3, ABCG5, multidrug resistance-associated proteins (MRP) type 1 (MRP1/ABCC1), and bile salt export pump (BSEP/ABCB11) are known to be important for maintaining BA homeostasis in the human liver (Gerloff et al., 1998; Pradhan-Sundd and Monga, 2019) and intestine (Berge et al., 2000; König et al., 2000), but their role in the placenta is still unclear. BA transporters that were consistently shown to be expressed in the placenta and are assumed to be involved in the transcellular transfer of BA are discussed in the following sections.

#### 2.3.1 Pathways of bile acid transport at the apical syncytiotrophoblast layer

The ABC transporters BCRP/ABCG2, MDR1/ABCB1, MRP2/ABCC2, MRP3/ABCC3, MRP4/ABCC4, the solute carriers OATP4A1/SLCO4A1 and ASBT/SLC10A2, and the P-type ATPase FIC1/ATP8B1 are reported to be localized at the apical side of the placental syncytium (Ontsouka et al., 2021) which is consistent with their role in the retrograde transport towards the maternal compartment (Figure 2). Many of the apical transporters mentioned above have partially overlapping substrate specificities. Substrates include cholesterol, nutritional compounds, xenobiotics, different BA species (e.g., taurocholate and cholate), estrogens and prostaglandins and their sulfated derivates (Allikmets et al., 1998; Zelcer et al., 2003; Lam et al., 2005). For some of these transporters consistent expression throughout gestation has been found in the human placenta. Depending on the study, *ABCG2* gene expression has been shown to both increase and remain unchanged across gestation (Mathias et al., 2005; Meyer Zu Schwabedissen et al., 2005; Yeboah et al., 2006; Petrovic et al., 2015; Sieppi et al., 2016). The role of placental ABCG2 and MRP3 in the increase of maternal serum BAs was recently confirmed (Liu et al., 2021) implying a protective function of these transporters for the fetus. ABCB1 (both mRNA and protein) has been reported to be highly expressed in the first trimester compared to term placentas (Mathias et al., 2005). In rats, placental mRNA expression of *Oatp1b2* decreased from gestational day 13 to day 20, then increased again immediately before birth, while the opposite pattern was reported for placental *Oatp-4* mRNA levels (St-Pierre et al., 2004). In contrast, *ATP8B1* mRNA expression was significantly reduced (33-fold) in the third compared to the first trimester in humans (Patel et al., 2003). In general, these data suggest that the investigated transporters are involved in the maintenance of maternal-fetal BA homeostasis at different gestational stages.

#### 2.3.2 Pathways of bile acid transport at the basal syncytiotrophoblast layer

The ABC transporter MDR3/ABCB4 as well as the solute carriers OATP2B1/SLCO2B1, OATP3A1/SLCO3A1, NTCP/SLC10A1, OST-α/SLC51A1/, OST-β/SLC51B1, and the small protein mEH/EPHX1 were reported to be located at the basal side of the syncytiotrophoblast layer (Ontsouka et al., 2021). Given their transport directionality and specificity, these proteins modulate fetal exposure to a broad range of substrates, including nutritional compounds, sulfated steroids, and BA species (Dippe et al., 1996; Tamai et al., 2000; Huber et al., 2006; Roth et al., 2012). *ABCB4* mRNA expression in the human placenta was increased 4-fold in the third trimester compared to the first trimester (Patel et al., 2003). Considering the unidirectionality of the substrate transport by ABCB4 towards the fetal circulation (Figure 2), its gestation-dependent increase may suggest an incremental role of ABCB4 in sustaining fetal development. ABCB4 is potentially also important in transporting BAs to their sites of receptor-mediated actions in the developing fetus as well as in the placenta itself. Moreover, given the BA transport directionality suggested for NTCP*,* OATP2B1, OATP3A1, OST-α/-β, and mEH in an earlier study (Ontsouka et al., 2021), these transporters may represent active players in the retrograde BA transport system towards the placenta/mother, thus protecting the fetus from overexposure to BA.

#### 2.3.3 Bile acid transport in intrahepatic cholestasis of pregnancy

The most common BA-related disorder specific to pregnancy is intrahepatic cholestasis of pregnancy (ICP), a disease that can occur as early as the seventh week of gestation but appears more frequently in the third trimester. It is characterized by high levels of maternal BA concentrations with a cut-off value of 10 μmol/L (Manzotti et al., 2019) and up to a maximum value of 430 μmol/L (Lammert et al., 2000). ICP is clinically characterized by pruritus, abnormal liver function tests (liver transaminases), and supra-physiological levels of circulating BAs (Lammert et al., 2000). There is a well-documented correlation between increased maternal blood BA levels and increased rates of fetal complications. In cases of severe ICP, where maternal serum BA concentrations reach 100 μmol/L (Brouwers et al., 2015), a poor pregnancy outcome is expected, including preterm labor (Geenes and Williamson, 2009), fetal distress, fetal asphyxia, and even intra-uterine death (Fisk and Storey, 1988; Glantz et al., 2004; Williamson et al., 2004). In these cases, greater concentrations of BAs are detected in the amniotic fluid, similar to women who gave birth to infants suffering from prenatal intestinal obstruction (Délèze et al., 1977).

Mutations in some of the apical efflux transporters have been associated with pregnancy-related diseases (Jacquemin et al., 1999; Müllenbach et al., 2005; Aydın et al., 2020). As suggested by (Müllenbach et al., 2005; Aydın et al., 2020), mutations in ATP8B1/FIC1 were found to be linked to ICP in humans. This aminophospholipid translocase modulating membrane asymmetry (Tang et al., 1996) was speculated to cause or contribute to ICP, but it is still unclear how mutations in the translocase activities of ATP8B1/FIC1 (Müllenbach et al., 2005; Aydın et al., 2020), may be mechanistically involved in this disease. Clinical treatment of ICP patients using UDCA has been associated with alterations in the placental expression of some BA transporters. One study reported a significant increase in the placental expression of MRP2 (both protein and mRNA) while MRP3 protein levels were not significantly different in ICP patients treated with UCDA compared to controls (Azzaroli et al., 2007). Similarly, UDCA treatment in humans was associated with significant upregulation of placental mRNA and protein levels of ABCG2 (Azzaroli et al., 2013). On the other side, in patients with moderate ICP which were treated with UDCA, neither the mRNA expression of ABCG2 nor MRP2 was significantly different compared to controls (Ontsouka et al., 2021). Thus, the discrepancy regarding the MRP2 gene expression between the latter two studies (Azzaroli et al., 2007; Ontsouka et al., 2021) may be linked to the variable severity degree found in the ICP patients. Finally, an association between a mutation in the ABCB4 gene (located on the basal side of the syncytiotrophoblast layer) and the occurrence of ICP was also documented (Jacquemin et al., 1999), suggesting its possible mechanistic involvement in the pathogenesis of ICP. Similarly, treatment with UDCA was associated with significant downregulation of *SLCO3A1* mRNA in ICP patient treated with UDCA as compared to controls (Ontsouka et al., 2021). A model summarizing the molecular mechanisms through which BAs interact with their receptors and transporters at the apical and basal sides of the syncytiotrophoblast layer is shown in Figure 2.

### 2.4 Role of the placenta in bile acid homeostasis

#### 2.4.1 The entero-hepato-placental axis: its influence on circulating serum bile acids

It is well established that the enterohepatic circuit determines serum BA levels (Hofmann, 2009). So far, the placenta has been primarily considered as a physical barrier between the maternal and fetal compartments, across which various substances including BAs and gases are exchanged (Herraez et al., 2014; Liu et al., 2021), thereby influencing the levels of these compounds in the maternal and fetal blood. Noticeably, the placenta is also a steroidogenic organ capable of the synthesis of diverse hormones such as progesterone using cholesterol as a precursor molecule (Karahoda et al., 2021). Interestingly, there is evidence that also extrahepatic tissues (e.g., the brain) are capable of synthesizing BAs and therefore contribute to the serum BA pool (Pan et al., 2017; Monteiro-Cardoso et al., 2021). In the context of pregnancy, previous studies have shown that serum BA levels are higher in pregnant compared to non-pregnant women and mice (Milona et al., 2010), regardless of the gestational age (Colombo et al., 1985; Egan et al., 2012). The concentration of BAs in healthy pregnancies is usually higher in the fetal blood than in the maternal circulation (Itoh et al., 1982; Colombo et al., 1985). In this physiological situation, the placenta serves as an exchange interface that prevents the accumulation of BAs in the fetal compartment to avoid deleterious effects on the fetus (Sewell et al., 1980; Colombo et al., 1985; St-Pierre et al., 2000). However, a study by Sasaki et al. reported that in 52.6% of the patients the BA content in the maternal serum was higher than in the umbilical cord, while in 36.8% of the cases the opposite was observed, and in 9.2% the BA content was similar (Sasaki, 1984). Overall, the vectorial transfer of the primary BAs across the placenta occurs mainly from the fetus to the mother (Figure 2), although secondary maternal BAs are transported from the mother into the fetal vessels (Colombo et al., 1987). A recent study performed in rats further suggested that also sex may play a role in BA concentrations since the total amount of placental BAs was two times higher in males than in females, but the opposite trend was observed in the fetal serum (Huang et al., 2021). Hence the factor sex adds another important variable influencing the selective allocation of BAs on both sides of the placental interface.

Given the central role of the placenta in facilitating the bi-directional transport of BAs (Figure 2), it is not surprising that placental dysfunction is a hallmark accompanying pregnancy-related metabolic diseases (e.g., preeclampsia, ICP). In ICP, particularly in severe cases (Brouwers et al., 2015), BAs are thought to be transported from the mother to the developing fetus (Meng et al., 1997; Tribe et al., 2010; Geenes et al., 2014; Liu et al., 2021; Ontsouka et al., 2021) exposing it to supraphysiological and toxic levels (Figure 2). Given the fetal over-exposure to BAs in ICP, one may question whether the placenta acts as an efficient BA protective barrier or rather as an exchange interface. It is important to note that elevated serum BAs in pregnant women do not necessarily imply ICP, since a subset of healthy pregnant women spontaneously presents levels above the mentioned cut-off value - a condition called asymptomatic hypercholanemia of pregnancy (AHP) - without suffering from ICP-related complications or other liver diseases (Pascual et al., 2002; Castaño et al., 2006). In this context, one study comparing the profiles of serum BAs between ICP and normal pregnancies including AHP suggested a shift towards a hydrophobic composition and free BAs in women with ICP (Castaño et al., 2006). This implies that the deleterious effects commonly attributed to this disease may result from the BA *profile* rather than the total *amount* of BAs. Moreover, it has been reported that some BA species —especially hydrophilic BAs such as UDCA and its taurine-conjugated derivative— exhibit beneficial health effects. Among others, they promote cytoprotective effects by inhibiting cellular oxidative stress and apoptosis (Perez and Britz, 2009). Serum BA profiles on both sides of the placenta are markedly different in healthy pregnancies (Sasaki, 1984; Geenes et al., 2014), highlighting the potential involvement of the placenta in the selective distribution of BA species between the maternal and fetal blood. Sasaki et al. (Sasaki, 1984) compared the BA distribution in maternal and umbilical cord sera in healthy pregnancies and showed that GCDCA was more prominent in the mother, while TCDCA was predominant in the umbilical cord (Sasaki, 1984). In contrast, the most abundant species in the fetal circulation in ICP is CA, which accounts for approximately 70%–80% of all circulating BAs (Tribe et al., 2010; Geenes et al., 2014). The observed differences imply an important role of the placenta in the transport, selection, and metabolism of the BAs arriving from both the mother and the fetus. Moreover, the placenta potentially complements the arriving BA pool with own locally synthesized BAs given its intrinsic placental steroidogenic machinery (see chapter 2.4.2.).

#### 2.4.2 The placenta as a possible source of bile acids

**FIGURE 3:**
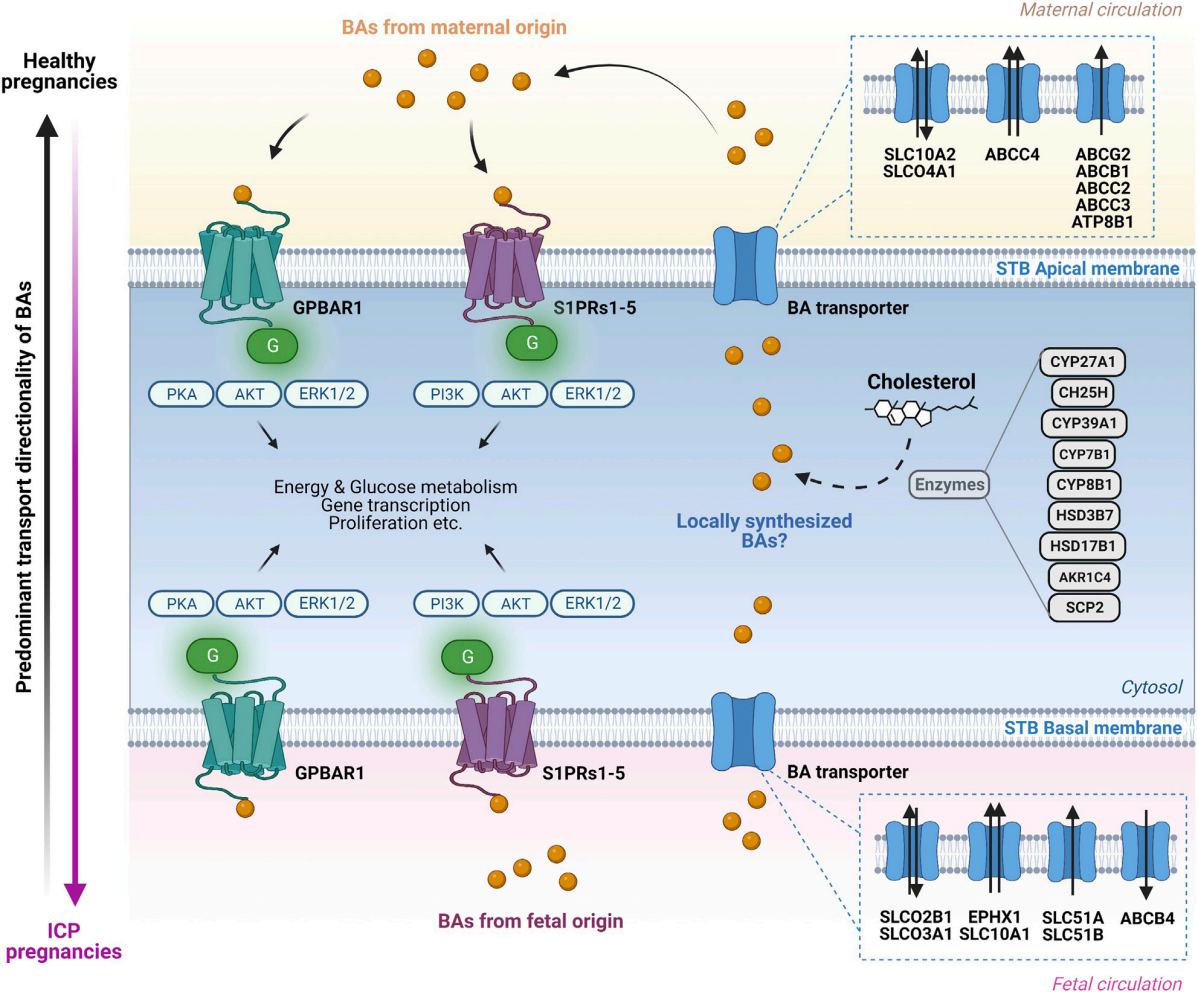
Bile acid transport and signaling pathways in the human placenta. Overview of the molecular mechanisms through which bile acid (BA) transporters and receptors operate at the apical and basal side of the syncytiotrophoblast (STB) layer. On the maternal side (top) BAs interact in an endocrine and probably autocrine/paracrine manner with BA-specific cell surface receptors located on the apical STB membrane. The BA pool in the maternal circulation consists predominantly of primary and secondary BAs. Additional BAs originating from fetal synthesis as well as from potential local synthesis by the placenta are added to the BA pool. BA are transported in both directions across the apical STB layer by specific transporters. At the fetal side of the STB (bottom), BAs interact in an endocrine and possibly autocrine/paracrine manner with specific cell surface receptors located at the basal STB membrane. The BAs in the fetal compartment are a mix of fetal-originating BAs as well as BAs transported across the basal STB membrane originating from the maternal circulation and potentially from local placental synthesis. Within the STB, BAs interact with BA-sensitive nuclear receptors (not depicted) with functional consequences on BA homeostasis (modulation of BA transporters, receptors, and potentially BA synthesis-related enzymes expression). The transport of BAs is primarily mediated by anion/BA exchangers at the basal (fetal) side of the trophoblasts (SLCO2B1, SLCO3A1, SLC51A and SLC51B), whereas the retrograde transport from the trophoblasts towards the mother occurs primarily via ABC transporter proteins (ABCG2, ABCB1, ABCC2, ABCC3 and ABCC4). The predominant direction of BA transport in healthy pregnancies occurs from the fetus to the mother (vertical black arrow, left) while in intrahepatic cholestasis of pregnancy (ICP) there is exacerbated BA transport from the mother to the fetus, exposing the latter to toxic levels (vertical pink arrow, left). Figure created with BioRender.com.

In a recent study, we reported the presence of mRNA transcripts of most of the BA synthesizing enzymes in the placentas of healthy mice and humans and proposed this organ as a potential new source of BA (Ontsouka et al., 2023). In this study, a species-specific expression profile of BA synthesizing enzymes was observed in placental tissues. Among others, *CYP7A1, CYP46A1*, and *BAAT* were undetected in human placental tissue, but their homologs were detected in the mouse placenta. Conversely, mRNA of *Cyp8b1* and *Hsd17b1* were missing in the mouse placenta, while their homologs were expressed in the human placenta. *CYP39A1/Cyp39a1* and cholesterol 25-hydroxylase (*CH25H/Ch25h*) mRNA were detected in both species. It should be also noted that, even though mRNA transcripts of *CYP7A1* were not consistently detected in the human placenta, they were found in isolated primary cytotrophoblast and syncytiotrophoblast cells. The apparent discrepancy between placental tissues and cell-based data could have resulted from a dilution effect occurring in placental tissue, which contains a myriad of cell types. The findings reported by (Ontsouka et al., 2023) are in line with studies where another extrahepatic organ —the brain —was found to exhibit BA biogenetic capabilities (Pan et al., 2017; Monteiro-Cardoso et al., 2021). To date, the majority of studies focused on investigating the role of the placenta exclusively as an interface across which BAs are transported between the mother to the fetus (Sewell et al., 1980; Itoh et al., 1982; Colombo et al., 1985; Meng et al., 1997; St-Pierre et al., 2000; Tribe et al., 2010; Geenes et al., 2014; Liu et al., 2021; Ontsouka et al., 2021). Available data at https://www.proteinatlas.org, supplemented with mRNA data from (Ontsouka et al., 2023), are compiled in Table 1 and provide indications regarding the potential capacity of the human placenta to synthesize BAs. For most of the enzymes involved in BA biosynthesis, an overlap exists between their expression profiles in the human liver, brain, and placenta (Table 1). However, it should be considered that the BA enzymatic machinery has been described so far only at the mRNA level (Ontsouka et al., 2023) and confirmation at the protein level is still lacking. At this stage, it is important to decipher whether primary and secondary BAs that cross the placenta are only of hepatic/intestinal origin or originate *in situ* in the placenta because BAs locally synthesized in the placenta may directly affect placental performance and fetal development (Sepúlveda et al., 1991; Egan et al., 2012; De Aguiar Vallim et al., 2013; Lofthouse et al., 2019; Li et al., 2020) (Figure 3). Considering both the recent molecular evidence implying potential placental BA biogenesis (Ontsouka et al., 2023), and the fact that serum BA levels are higher in pregnant compared to non-pregnant women and mice (Milona et al., 2010), the previous belief that solely the enterohepatic axis determines the serum concentration of BAs (Hofmann, 2009) should be re-evaluated. We propose that serum concentrations of BAs during healthy pregnancies (including AHP) are probably maintained thanks to the contribution of all three compartments (mother-placenta-fetus) of the entero-hepato-placental axis (Figure 3). Although the fetal liver and the placenta may contribute to the circulating BA levels within the entero-hepato-placental axis in addition to the maternal organs (Figure 3), their respective relative contributions must still be elucidated.

**TABLE 1:** Localization of bile acid synthesis-related enzymes in hepatic and selected extrahepatic human tissues.

		Selected sites of enzyme synthesis		
Abbreviation	Name	Liver	Brain	Placenta
CYP7A1	Cholesterol 7-hydroxylase	HepC	-	STB, CTB
CYP46A1	Cholesterol 24-hydroxylase	HepC, KupC, EndC	Neu, OligC, AstrC	EndC, FibC, HofC, CTB
CH25H	Cholesterol 25-hydroxylase	KupC, EndC	MicC, Neu	HofC, EndC, FibC, STB, CTB
Diiron factor				
CYP8B1	Sterol 12 alpha-hydroxylase	HepC	-	STB, CTB
CYP27A1	Sterol 27-hydroxylase	HepC, KupC	OligC, AstC, Neu	HofC, FibC, STB, CTB
CYP39A1	Oxysterol 7-hydroxylase	HepC, Ito cells, T-cells, KupC	MicC, OligC, AstC, Neu	EndC, ETB, FibC, HofC, STB, CTB
CYP7B1	Oxysterol 7-hydroxylase	HepC, Ito cells, T-cells, KupC	MicC, OligC, AstC, Neu	FibC, EndC
C27 3β-HSD, SDR11E3	Hydroxy-δ5-steroid dehydrogenase, 3 beta- and steroid delta-isomerase 7	HepC, T-cells, KupC, ChoC	AstC, OligC, Neu	HofC, FibC, EndC, STB, CTB
AKR1D1	Delta-4-3-Oxosteroid 5β-reductase	HepC, T-cells?	-	HofC, CTB, EndC
AKR1C4	3α-Hydroxysteroid dehydrogenase	HepC	-	STB, CTB
hVLCS-H2 (SLC27A5)	Bile acid-CoA ligase	HepC	AstC, OligC, Neu	HofC, FibC, CTB, EndC
BAAT	Bile acid-CoA: amino acid N-acyltransferase	HepC, ChoC	AstC, OligC, Neu	-
AMACR	Alpha-methylacyl-CoA racemase	HepC, ChoC, KupC, EndC, T-cells	-	CTB, HofC, EndC, FibC
ACOX2	Branched-chain acyl-CoA oxidase	HepC, KupC, EndC	AstC, Neu	FibC, HofC, CTB, EndC
HSD17B1	D-Bifunctional protein	HepC, ChoC, KupC, EndC	AstC, OligC, Neu	STB, CTB
SCPx	Sterol carrier protein X (Peroxisomal thiolase)	HepC, KupC, EndC	AstC, OligC, Neu	STB, CTB, FibC, HofC, EndC

AstC: astrocytes; ChoC: cholangiocytes; CTB: cytotrophoblasts; EndC: endothelial cells; FibC: fibroblasts; HepC: hepatocytes; HofC: Hofbauer cells; KupC: Kupffer cells; MicC: microglial cells; Neu: neurons; OligC: oligodendrocytes; STB: syncytiotrophoblasts. Data are taken from https://www.proteinatlas.org and derived from own mRNA analyses in primary trophoblasts as published in (Ontsouka et al., 2023). The symbol ‘-’ means that the expression is not reported.

## 3 Summary and conclusion

The present manuscript discusses the physiological importance of BAs as signaling molecules and reviews the membrane proteins and mechanisms that sustain the transport of BAs across the placenta, which subsequently contributes to the BA levels in fetal and maternal circulations. Furthermore, the placenta may act not only as an exchange interface but may also serve as a steroidogenic organ capable of synthesizing BAs. These placentally synthesized BAs are possibly added to the maternal and fetal BA pools and may additionally exhibit receptor-mediated autocrine and paracrine effects. Although additional investigations are needed to unequivocally ascertain the capacity of the placenta to synthesize BAs, this possibility opens avenues to explore the importance of *in situ* BA secretion on the regulation of placental performance, fetal organ growth and maturation, as well as fetal programming of various vulnerabilities.
